# Interpersonal Conflict, School Connectedness and Depressive Symptoms in Chinese Adolescents: Moderation Effect of Gender and Grade Level

**DOI:** 10.3390/ijerph16122182

**Published:** 2019-06-20

**Authors:** Guan-Hao He, Esben Strodl, Wei-Qing Chen, Fan Liu, Alimila Hayixibayi, Xiang-Yu Hou

**Affiliations:** 1 Department of Biostatistics and Epidemiology, School of Public Health, Sun Yat-sen University, Guangzhou 510080, China; heguanh@mail2.sysu.edu.cn (G.-H.H.); alimila.hayixibayi@hdr.qut.edu.au (A.H.); 2 School of Psychology and Counselling, Queensland University of Technology, Brisbane, QLD 4059, Australia; f6.liu@connect.qut.edu.au; 3 Department of Information Management, Xinhua College of Sun Yat-Sen University, Guangzhou 510520, China; 4 School of Public Health and Social Work, Queensland University of Technology, Brisbane, QLD 4059, Australia; x.hou@qut.edu.au

**Keywords:** depressive symptoms, interpersonal conflict, school connectedness, Chinese adolescents, gender, grade level

## Abstract

This study examined the associations of interpersonal conflict and school connectedness with depressive symptoms in Chinese adolescents. A cross-sectional study was conducted among 6576 adolescents in Shenzhen, China. Participants completed a battery of questionnaires that assessed adolescents’ depressive symptoms, conflict with parents, teachers, and peers, school connectedness, and demographics. Multiple linear regression analysis was used to explore the association of interpersonal conflict and school connectedness with depressive symptoms in adolescents. Results showed that conflicts with their mother, father, teachers, and peers were associated with higher levels of depressive symptoms in adolescents, while greater school connectedness was related to lower levels of depressive symptoms in adolescents. Gender proved to be a moderator of these relationships in that the associations of quarreling with mothers, mothers’ use of emotional punishments, teachers’ use of emotional punishments, and school connectedness with depressive symptoms were stronger in females than males. Moreover, grade level proved to be another moderator, with the associations of teachers’ use of physical punishment and fighting with peers with depressive symptoms being stronger in primary school students than in secondary school students. Our findings suggest that gender and grade level moderated the association of interpersonal conflict and school connectedness with depressive symptoms in Chinese adolescents.

## 1. Introduction

Depression is a common mental health condition with the probability of onset increasing markedly with puberty compared with childhood [1]. Epidemiology studies have indicated that adolescents with higher levels of depressive symptoms tend to encounter increased risk of adverse events such as poor academic performance [2], higher levels of substance use [3], and higher levels of suicide [4]. Furthermore, a cost-of-illness study showed that depression in adolescents can cause tremendous societal costs to families [5]. Given that, there is a pressing need to better understand the risk factors for adolescent depression in order to guide the development of effective public health interventions.

While acknowledging biological risk factors for depression such as genetic factors, environmental factors also play a significant role in the development of adolescent depressive symptoms [1]. One critical environmental factor is family interpersonal relationships. For instance, Auerbach et al. reported that family conflict was prospectively related to greater depressive symptoms among Canadian adolescents [6]. Fosco et al. also found that increasing levels of family conflict during middle school was associated with higher levels of depressive symptoms in adolescents [7]. However, previous studies largely focused on the overall family conflict in Western countries, while little consideration has been given to the specific influence of mother-adolescent conflict and father-adolescent conflict in Asian countries like China. Previous studies have indicated that Chinese Confucianism culture emphasizes obedience to parents and elders [8] and Chinese mothers have been reported to apply more corporal punishment and physical restraint than American mothers [9]. Given that the level of parent-child conflict varies across culture [10], as well as the scarce evidence within Chinese culture, the current study aimed to examine mother-adolescent conflict and father-adolescent separately, and to explore their specific associations with depressive symptoms in adolescents.

In addition to family interpersonal relationships, school based interpersonal relationships such as those between adolescents and teachers and peers are also important risk factors for adolescent depression. Regarding the influence of teacher-student relationships, Mizuta et al. found that higher teacher support was related to lower levels of depressive symptoms among Japanese adolescents [11]. In addition, a longitudinal study conducted in China also indicated that teacher support in 7th grade was associated with lower levels of students’ depression and anxiety in the 8th grade [12]. While these studies look at the relationship between the presence of supportive teacher-student relationships and depression, interestingly little is known about the presence of conflictual teacher-student relationships and depression symptomatology. There is evidence however that conflictual teacher-student relationships may be associated with other measures of mental health. For example, Lee et al. demonstrated that teacher-student conflict in the early elementary years could positively predict students’ aggression in 5th grade [13]. Similarly, a longitudinal study conducted by Skalicka and colleagues found that teacher-student conflict was associated children’s concurrent and subsequent externalizing behaviors [14]. In addition, previous study has demonstrated that Chinese teachers tend to use less punishment and aggressive behaviors towards students than Australian and Israeli teachers [15]. Given that, there is a need to further explore whether conflictual teacher-student relationships may be risk factors for the presence of Chinese adolescent students’ depressive symptoms.

With respect to the influence of peer relationships, a study conducted among British adolescents demonstrated that peer victimization was associated with higher levels of concurrent and subsequent depressive symptoms [16]. Similarly, associations between peer victimization and levels of depressive symptoms have been found in Korean adolescents [17] and in Hong Kong adolescents [18]. Based on these findings, our study aimed to examine these findings in a Chinese mainland population, and build upon this research by exploring the association between the specific types of peer conflict and depression in adolescents. Moreover, by simultaneously measuring adolescent conflict with parents, teachers, and peers, it allows us to gain a more comprehensive understanding of the role of a range of interpersonal conflicts upon depression in adolescents.

In addition to interpersonal conflict in school, school connectedness is another useful measure of interpersonal relationship in schools. School connectedness refers to the belief by students that adults and peers in the school care about their learning as well as about them as individuals [19]. The inclusion of school connectedness can enhance our investigation into school-based relationships by examining the presence of positive relationships as well as the presence of negative or conflictual relationships. School connectedness has been reported to be negatively associated with depressive symptoms in adolescents in western countries [20]. More relevant to our study, Zhao et al. found that school connectedness was negatively correlated with depression in Chinese adolescents [21]. Nevertheless, the narrow age range in this study (16–18 years) restricts a better understanding of the influence of school connectedness on anxiety in a broader range of Chinese adolescents. The present study thus aimed to examine this association in Chinese adolescents aged from 10 to 18 years old.

This study therefore aimed to understand the relationships between parent-adolescent conflict, teacher-adolescent conflict, peer-adolescent conflict, and school connectedness and depressive symptoms in Chinese adolescents. Given that the level of depressive symptoms in adolescents varies across gender and grade level [22], there is an implication that gender and grade level may be moderators of association of predictors such as interpersonal conflict and school connectedness with depressive symptoms in adolescents. As such this study additionally aimed to examine the moderation effects of gender and grade level on these associations among Chinese adolescents. More specifically given that there is strong evidence through a meta-analysis that the association between social support and adolescents’ well-being is stronger in females than males [23], we predict that the associations between interpersonal conflict and school connectedness with depressive symptoms will be stronger in female adolescents than male adolescents. In addition, given that the association between social support and emotional well-being in adolescents increases with age [23], we predict that the associations between interpersonal conflict and school connectedness will be stronger in secondary school students than in primary school students. As such the hypotheses of the present study are as follows:

(1) Parent-adolescent conflict will be associated with higher levels of depressive symptoms in adolescents.

(2) Teacher-adolescent conflict will be related to higher levels of depressive symptoms in adolescents.

(3) Peer-adolescent conflict will be related to higher levels of depressive symptoms in adolescents.

(4) Higher levels of school connectedness will be associated with lower levels of depressive symptoms.

(5) Gender and grade level will serve as moderators in the above associations. The strength of these associations will be stronger among female adolescents than males, and will be stronger in adolescents who are in secondary school compared with adolescents in primary school.

## 2. Materials and Methods

### 2.1. Study Design and Participants

A school-based, cross-sectional study with random stratified cluster sampling was used to obtain the participants in Longhua District, Shenzhen, China. In stage 1, a random cluster sampling was used, and 24 out of the 59 schools were selected, including primary schools (grade 1 to 6 with children aged around 6 to 12) and secondary schools (grade 7 to 12 with adolescents aged around 13 to 19). In stage 2, three classes were randomly chosen from each grade of grades 5, 6, 7, 8, 10 and 11 from the 24 schools. All available students in the selected classes were invited to participate in our study. A total of 6638 students were invited to participate in the study, and 6584 students agreed to participate and completed the questionnaire with a response rate of 99.2%. The students or their parents who refused to participate in this study were excluded from the analysis. According to the WHO’s standard for adolescent age (10–19 years old) [24], eight students were excluded due to having ages outside of the inclusion criteria range leaving 6576 adolescents in the final analysis.

### 2.2. Data Collection

The survey was conducted in the classroom during class time. The students were asked to fill in questionnaires concerning the demographics of students and their parents, students’ ranking in class, students’ conflict with parents, teachers, and peers, students’ school connectedness and students’ depressive symptoms. The questionnaires were administrated by the members of the research team in the classroom without the presence of teachers. All questionnaires were anonymous to protect the privacy of the students. Students who were not willing to participate were instructed that they could hand in blank questionnaires at the end of survey so as not to identify themselves. All data were collected in 2015.

### 2.3. Ethical Statement

This study was approved by the Ethics Committee of the School of Public Health at Sun Yat-sen University, Guangzhou, China (2015–2016). All the participants were completely informed of the purpose of the study, and they were invited to join voluntarily. Written informed assent was obtained from all participating students, while written informed consent was provided by the students’ parents.

### 2.4. Measures

#### 2.4.1. Demographic Factors

Demographic variables measured in this study included gender, age, grade level (primary school, secondary school), participant’s academic ranking in the class (1–10, 11–30, >30), parents’ ages, and parents’ educational levels (≤9th grade, ≤high school, and ≥undergraduate).

#### 2.4.2. Depressive Symptoms

The Depression Self-Rating Scale for Children (DSRSC) was used to measure depressive symptoms in participants. The DSRSC, developed by Birleson in 1981, is an 18 item validated self-reported screening tool to assess depression in children and adolescents [25]. Participants rate each item on a three-point Likert scale: 0 = never, 1 = sometimes, and 2 = often. The items are summed up to get a total score ranging from 0 to 36. This scale was translated into Chinese in by Su et al. and proved to have fair internal reliability (Cronbach α = 0.73) and good validity [26]. As such this translated scale has been widely used in epidemiology and psychology studies among Chinese adolescents [27,28]. The Cronbach’s α for the DSRSC was similar and acceptable in our study at 0.67. The total score of DSRSC was used as a measure of depressive symptoms in our study.

#### 2.4.3. Conflicts with Father and Mother

Three common types conflict with father and mother were measured in the current study. These questions were selected from a previous study conducted in Chinese adolescents [29]. The three individual questions concerning conflict with the father were: “Have you ever had a serious quarrel with your father in the past 12 months?”, “Have you ever been emotionally punished (such as being scolded, threatened, or humiliated) by your father in the past 12 months?” and “Have you ever been physically punished (such as being forced to stand for some time, being beaten with fist or other objects, or being kicked) by your father in the past 12 months?” Similarly, three identical questions were used to measure conflict with mother.

#### 2.4.4. Conflicts with Teachers

Three common types conflict with teachers were also measured. These questions were also drawn from the study by Sun et al. which was conducted among Chinese adolescents [29]. The three individual questions concerning conflict with teachers included: “Have you ever had a serious quarrel with your teacher in the past 12 months?”, “Have you ever been emotionally punished (such as being scolded, threatened, or humiliated) by your teacher in the past 12 months?” and “Have you ever been physically punished (such as being forced to stand for some time, being beaten with fist or other objects, or being kicked) by your teacher in the past 12 months?”.

#### 2.4.5. Conflicts with Peers

Similarly based upon previous research with Chinese adolescents [29], three types of common conflicts with peers were assessed with three individual items: “In the past 12 months, have you had a serious quarrel with your fellow students at school?”, “In the past 12 months, have you been involved in physical fighting with your fellow students at school?” and “In the past 12 months, have any of your fellow students emotionally bullied you (humiliated, teased, or threatened you)?”

#### 2.4.6. School Connectedness

The level of school connectedness was measured using a five-item scale, which was derived from the National Longitudinal Study of Adolescent Health School connectedness scale [30]. Participants were asked to answer how strongly they agree or disagree with five statements concerning school connectedness, including: “I feel safe in my school”, “The teachers at this school treat students fairly”, “I am happy to be at this school”, “I feel like I am part of this school”, and “I feel close to people at this school”. Each item was rated on a five-point Likert scale: 0 = strongly disagree, 1 = disagree, 2 = neutral, 3 = agree, 4 = strongly agree. The responses were reverse-coded and then summed up, with a total score ranging from 0 to 20. Higher scores indicated greater school connectedness. This scale is a unidimensional measure which has been shown to have good reliability (Cronbach α from 0.82 to 0.88) and validity across 18 sociocultural groups (including Chinese American adolescents) [31]. In addition, this scale was also used among mainland Chinese adolescents and proved to have good internal consistency (Cronbach α = 0.83) [29]. In the current study, the internal consistency was also found to be good at 0.84.

### 2.5. Statistical Methods

The current analysis involved 6576 adolescents. Among these participants, the percentage of missing data for all variables was less than 10%, except for maternal age (10.5%) and paternal age (11.8%). In addition, the amount of participants with complete data on all variables in the current study was 4833 (73.5% of 6576). Missing values were imputed using multiple imputations [32,33]. In accordance with the suggestion that multiple imputation requires 20 imputations for 30% missing information [34], we created 20 data sets where the missing data in the original data set were imputed. All variables were used as predictors in the multiple imputation. Then the 20 imputed data sets were analyzed using standard statistical analyses, and the results from these 20 complete data sets were combined to produce inferential results.

Continuous variables were described as mean (SD) and categorical variables were described as frequencies and proportions. Depressive symptoms and school connectedness were compared between different genders and grade levels by *t*-test. Interpersonal conflict between different genders and grade levels were compared by *χ*^2^ test. The variables with multiple categories were transformed into dummy variables before further analyses. Multiple linear regression was applied to explore the association of interpersonal conflict and school connectedness with depressive symptoms in adolescents (represented by the total score of DSRSC), after adjusting for the covariates. The covariates included adolescents’ gender, grade level, ranking in class, maternal age, maternal education level, paternal age, and paternal education level.

In order to further examine the potential moderation effect of gender, an interaction item, namely interpersonal conflict (or school connectedness) × gender, was added in the above regression model. When the interaction item was significant, we conducted further subgroup analysis to explore the specific associations of interpersonal conflict and school connectedness with depressive symptoms in adolescent males and in adolescent females. Similarly, to detect the moderation effect of grade level, an interaction item, namely interpersonal conflict (or school connectedness) × grade, was also added in the regression model. When the interaction item was significant, we conducted further subgroup analysis among adolescents in primary schools and adolescents in secondary schools to evaluate the specific associations separately.

All statistical analyses were applied with SAS 9.4 (SAS Institute Inc, Cary, NC, USA). Statistical tests were two-tailed with a significance level of *p* < 0.05.

## 3. Results

### 3.1. Descriptive Statistics

The demographic characteristics of the participants are shown in Table 1. In this sample, 58.0% were boys and the mean age was 13.37 years (SD = 1.84). Further, of the surveyed adolescents, 43.7% were in primary schools, 56.3% were in secondary schools. In terms of parental demographics, 11.3% of mothers and 16.8% of fathers had bachelor degree or above, and the mean age was 39.07 years (SD = 4.55) for mothers and 41.63 years (SD = 4.82) for fathers.

### 3.2. Depressive Symptoms Across Gender and Grade Level

Within the total sample, the mean score of depressive symptoms was 12.18 (SD = 5.39). In terms of gender difference, female adolescents had significantly higher levels of depressive symptoms than male adolescents (the mean was 12.70 vs. 11.75, *t*_(1, 6033)_ = −6.76, *p* < 0.001). In terms of grade difference, adolescents in secondary schools had significant higher levels of depressive symptoms than adolescents in primary schools (the mean was 12.63 vs. 11.57, *t*_(1, 6385)_ = −7.88, *p* < 0.001).

### 3.3. Interpersonal Conflict and School Connectedness across Gender and Grade Level

Table 2 shows the gender difference in interpersonal conflict and school connectedness. Compared to female adolescents, male adolescents encountered more quarrelling with their fathers, fathers’ use of emotional punishment, and fathers’ use of physical punishment. However, there was no gender difference in conflict with mothers. Moreover, male adolescents also tended to experience more quarrelling with teachers, teachers’ use of emotional punishment and teachers’ use of physical punishment. In addition, male adolescents experienced more quarrelling with peers, fighting with peers, and peers’ use of emotional bullying. Conversely, male adolescents reported lower school connectedness than female adolescents.

Table 3 shows the grade differences in interpersonal conflict and school connectedness. Adolescents in primary schools reported encountering less frequent quarrelling with their fathers and use of emotional punishment by their fathers than adolescents in secondary schools. Conversely, adolescents in primary schools experienced more frequent use of physical punishment by their fathers than adolescents in secondary schools. The same pattern of grade difference was also found in the conflict with mothers. In addition, adolescents in primary schools encountered less frequent quarrelling with teachers and use of emotional punishment by teachers than adolescents in secondary schools. There was no grade difference in teachers’ use of physical punishment. Moreover, compared to adolescents in secondary schools, adolescents in primary schools experienced more frequent quarrelling with peers, fighting with peers and use of emotional bullying by peers. Furthermore, adolescents in primary school reported a higher school connectedness than adolescents in secondary school.

### 3.4. Associations of Interpersonal Conflict and School Connectedness with Depressive Symptoms in Adolescents

Model 1 in Table 4 shows the results from the multiple linear regression that evaluated the associations of interpersonal conflict and school connectedness with depressive symptoms in adolescents after controlling for the covariates. In terms of father-adolescent conflict, every type of conflict with fathers (quarrelling with father, father’s use of emotional punishment, and father’s use of physical punishment), was related to greater levels of depressive symptoms in adolescents. In terms of mother-adolescent conflict, similar associations were found in that each type of conflict with the mother (quarrelling with mother, mother’s use of emotional punishment, and mother’s use of physical punishment) was associated with higher levels of depressive symptoms in adolescents.

With respect to teacher-adolescent conflict, when compared to the adolescents who had not experienced conflict with teachers (including all three types of conflict: quarrelling with teachers, teachers’ use of emotional punishment, and teachers’ use of physical punishment), those who had experienced conflict with teachers had a significantly greater level of depressive symptoms. With respect to peer-adolescent conflict, every type of conflict with peers (quarrelling with peers, fighting with peers, and peers’ use of emotional bullying) was related to higher levels of depressive symptoms in adolescents. As for school connectedness, higher levels of school connectedness were associated with lower levels of depressive symptoms.

### 3.5. Moderation Effect of Gender in the Associations of Interpersonal Conflict and School Connectedness with Depressive Symptoms in Adolescents

Interaction items between interpersonal conflict and gender (e.g., quarrel with father × gender), or between school connectedness and gender (e.g., school connectedness × gender) were added in the regression models to explore the moderation effects of gender (Model 2 in Table 4). When the interaction item was significant, subgroup analysis was conducted to explore the specific associations in males and females separately (Table 5). A significant positive interaction effect between adolescents quarrelling with their mother and gender upon depressive symptoms was found. After subgroup analysis across males and females, the association between quarrelling with their mother and depressive symptoms was found to be positive and significant in both males and females, however the absolute value of the *β* coefficient was larger in females than males. Similarly, the interaction effects between mother’s use of emotional punishment and gender, as well as between teachers’ use of emotional punishment and gender upon depressive symptoms were significantly positive. Subgroup analysis revealed that the absolute values of the *β* coefficients for these interpersonal conflicts were larger in females than males. Moreover, there was a significant interaction between school connectedness and gender. Subgroup analysis indicated that the absolute value of the *β* coefficient for school connectedness was also larger in females than males.

### 3.6. Moderation Effect of Grade Level in the Associations of Interpersonal Conflict and School Connectedness with Depressive symptoms in Adolescents

The interaction effects between interpersonal conflict and grade level (e.g., quarrel with father × grade), or between school connectedness and grade level (e.g., school connectedness × grade) were added in the regression models to explore the moderation effects of grade level (Model 3 in Table 4). A significantly negative interaction between teachers’ use of physical punishment and grade level upon depressive symptoms in adolescents was observed. After subgroup analysis, the association between teachers’ use of physical punishment and depressive symptoms was significant and positive in adolescents in both primary schools and secondary schools. However, the absolute value of the *β* coefficient for teachers’ use of physical punishment in primary school students was larger than the *β* coefficient in secondary school students. Finally, a significant interaction effect between fighting with peers and grade level upon depressive symptoms in adolescents was also observed, with the absolute value of *β* being larger among students in primary school than secondary school.

## 4. Discussion

This current study aimed to assess the associations of interpersonal conflict and school connectedness with depressive symptoms among Chinese adolescents. Further, we also aimed to examine the moderation effect of gender and grade level upon theses associations. The results of the study confirmed the first hypothesis that parent-adolescent conflict is associated with higher levels of depressive symptoms in adolescents. This finding is consistent with findings from some previous studies. For example, a longitudinal study among Canadian adolescents has revealed that more family conflict was prospectively related to greater depressive symptoms [6]. Fosco et al. also demonstrated that increasing levels of family conflict during middle school were related to higher levels of depressive symptoms in adolescents [7]. More relevant to our study, a research conducted among Korean adolescents found that family conflict was positively related to depression in adolescents [35]. While these researchers have shown significant association between family conflicts and depression in adolescents, this is the first study to differentiate between conflict with the mother or father and collect the information of different types of mother-adolescent conflict and father-adolescent conflict. Our study found that all three types of mother-adolescent conflict and father-adolescent conflict were related to greater depressive symptoms in Chinese adolescents.

These findings should be considered in terms of Chinese culture, based on Confucianism, which values collectivism and filial piety: cultural beliefs that emphasize obedience to norms and respect for parents and elders [8]. Some extreme parenting methods such as scolding and physical punishment may be used to achieve obedience and perceived respect to parents. Olson et al. indicated that Chinese mothers scored higher on harsh discipline (measuring corporal punishment, physical restraint, and public humiliation) [9]. If the frequency of parent-adolescent conflict is higher in Chinese families compared with other cultures, then our findings of association with depressive symptoms in adolescents strengthens the need to develop further public health interventions to reduce parent-adolescent conflict in China.

The second hypothesis that teacher-adolescent conflict would be related to higher levels of depressive symptoms in adolescents was also supported by our results. Consistently, previous studies have found that student-teacher conflict in childhood is associated with higher aggression [13] and externalizing behavior in western countries [14]. In addition, studies in Japan found that teachers’ support was independently related to lower levels of depression in adolescents [11]. As far as we are aware, our study is the first to find an association between student-teacher conflict and depressive symptoms in Chinese adolescents. Moreover, our study is the first study to demonstrate associations between three specific types of teacher-adolescent conflict (quarrelling with teachers, teacher’s use of emotional punishment, and teacher’s use of physical punishment) and depressive symptoms in Chinese adolescents. Again, these findings should be considered within the Chinese Confucian culture in that teachers have high social status and the respect of teachers is emphasized from a very early age. This makes the bond between teachers and students in China very significant to the student. Given that Chinese students report better teacher-student relationships than American students [36] and Chinese teachers use less punishment and aggressive behaviors with students than Australian and Israeli teachers [15], conflict with a teacher may be a relatively unusual experience with strong psychological consequences for Chinese adolescents. Therefore, further cross-cultural studies are required to better understand these associations identified in this study.

The third hypothesis that the peer-adolescent conflict would be related to higher levels of depressive symptoms in adolescents was also supported by our results. These findings were consistent with previous studies using measures of peer relationships conducted in other countries and regions. For example, there is evidence that peer victimization was related to higher levels of concurrent and subsequent depressive symptoms [16]. A study conducted among Korean adolescents revealed that students who had experienced peer victimization reported higher levels of depression [17]. More relevant to our study, a survey conducted in Hong Kong reported that peer victimization was positively related to depression [18]. As such, the current study builds upon these findings among mainland Chinese adolescents, and additionally highlights that all three specific types of peer-adolescent conflict (quarrelling with peers, fighting with peers, and peers’ use of emotional bullying) were related to greater depressive symptoms in adolescents.

Our results also supported our fourth hypothesis that higher levels of school connectedness would be associated with lower levels of depressive symptoms. Consistent with our findings, school connectedness has previously been found to be related to lower levels of depressive symptoms among Western (e.g., UK) adolescents [20]. A study conducted in China also revealed that school connectedness was related to lower levels of depression in adolescents aged 16 to 18 years [21]. As such, our study was based upon these studies and extended their findings to a broader age range that school connectedness was negatively associated with depressive symptoms in Chinese adolescents aged 10 to 18 years. In addition to depressive symptoms, other studies have demonstrated that adolescents who feel connected to their school are less likely to engage in many adverse events, including emotional distress, suicidality, violence, cigarette use, alcohol use, and marijuana use [30]. As such, there is a need to expand such research to other measures of mental health in Chinese adolescents.

To the best of our knowledge, this is the first study to explore the moderation effect of gender in the association of a series of interpersonal conflicts and school connectedness with depressive symptoms in adolescents. Regarding the final hypothesis, gender moderated the association of quarrelling with their mother and their mother’s use of emotional punishment with depressive symptoms in adolescents. The influence of these two types of mother-adolescent conflict on depressive symptoms were stronger in females than males. While other studies have not specifically examined conflict, previous studies support a similar moderating role of gender with evidence that the association between maternal relationship quality and depression in children and between maternal parenting attitudes and depression in adolescents was stronger in females than males [37,38]. A possible explanation is that gender role identification may make mothers a more crucial role for female adolescents to guide their feeling and behaviors, particularly during adolescence.

Our findings also indicated that gender moderated the association between teachers’ use of emotional punishment and depressive symptoms in adolescents. The influence of teachers’ use of emotional punishment on depressive symptoms was stronger in females than males. These results suggest that female adolescents may be more susceptible than male adolescents to teacher-adolescent conflict. A possible explanation is that there may be gender differences in student relationships with teachers. For example, Koepke and Harkins found that teachers reported higher conflict with boys than girls, while reporting higher levels of closeness with girls than boys [39]. Similarly, our finding also demonstrated that female adolescents reported less frequent use of emotional punishment by teachers than male adolescents. Given that female students might tend to experience and value closer relations with teachers than male students, it is possible that female students might experience higher levels of distress when there are perceived ruptures to these relationships via conflict.

In addition to these findings, gender also moderated the association between school connectedness and depressive symptoms in adolescents, with this association being stronger in females than males. Though few studies have explored this issue, there is evidence of gender differences in the association between school connectedness and adolescent suicide attempts, in that school connectedness was related to decreased suicide attempts in female adolescents but not in male adolescents [40]. More relevant to our study, Kendler conducted a longitudinal study among adults and found that social support was a stronger predictor of depression in females than males [41]. In addition, a meta-analysis conducted revealed that the association between social support and adolescents’ well-being was stronger in females than males [23]. These findings may be due to teenage girls relying more on interdependent relationships for their well-being [42].

As far as we know, this is the first study to explore the moderation of grade level in the association of interpersonal conflict and school connectedness with depressive symptoms in adolescents. Our findings demonstrated that grade level moderated the associations of teachers’ use of physical punishment and fighting with peers with depressive symptoms in adolescents. Interestingly, although the levels of depressive symptoms in adolescents were higher in secondary schools than those in primary schools, the influence of the teachers’ use of physical punishment and fighting with peers on depressive symptoms was stronger in adolescents in primary schools than those in secondary schools. These findings are contrary to our hypotheses and require further consideration. One possible consideration is that these findings may be due to adolescents’ ability to cope with stressors increasing with age [43]. That is, perhaps Chinese adolescents in secondary schools can manage the adverse events, such as teachers’ use of physical punishment and fighting with peers, better than those in primary schools. This finding requires further exploration in future studies.

While this study has a number of strengths, such as a large sample size and a high response rate, it also has some limitations. First, the current study design was a cross-sectional study where the data was collected concurrently, which restricted the ability to conduct causal inference. Longitudinal studies are needed to confirm the findings from this study. Second, it would have been helpful to have also assessed depression with a clinical diagnosis using a structured clinical interview. However, given the large sample size this was not feasible in our study. As such our findings are limited to understanding the relationships between interpersonal conflict and school connectedness with a self-reporting questionnaire measuring depressive symptoms. Third, the information about interpersonal conflict was self-reported by adolescents. Given that parents, teachers, and peers may have different perceptions of the interpersonal conflict compared to the participants, collecting this data from parents, teachers, and peers would allow a more comprehensive understanding of the relationship between interpersonal conflict and depressive symptoms in adolescents. Fourth, we did not collect information about adolescents’ Intelligence Quotient (IQ). Collecting this data would allow a more extensive understanding of the association of interpersonal conflict as well as school connectedness with depressive symptoms in adolescents. Fifth, we just collected the data of adolescents’ interpersonal conflicts rather than a comprehensive measurement of parent, teacher, and peer relationships with adolescents, which may limit our ability to explore the overall association between adolescents’ interpersonal relationships and their depressive symptoms.

As this is the first study to find the moderation effects of gender and grade level upon the associations of conflict with parents, teachers, and peers, and school connectedness with depressive symptoms in adolescents, there is a need for further studies to confirm these findings in other cultures. Associated with this there is a need for both prospective studies to clarify the direction of causality in these relationships across cultures, as well as for future studies to develop hypotheses about the cultural factors that impact these associations. Such deeper understanding of the causal relationships and the influence of cultural factors will help with the development of culturally sensitive and targeted interventions for adolescent depression.

## 5. Conclusions

Interpersonal conflict and school connectedness were found to have associations with levels of depressive symptoms in Chinese adolescents. In addition, gender and grade level moderated the associations of interpersonal conflict and school connectedness with the levels of depressive symptoms. Future prospective studies into adolescent depression should consider the inclusion of interpersonal conflict with parents, teachers, and peers, as well as school connectedness as possible predictors of depressive symptoms in adolescents. In addition, gender and grade level should be considered as possible moderators of these relationships.

## Figures and Tables

**Table 1:** Demographic characteristics of the participants.

Characteristic	No. Valid for Observation	No. Missing	Mean ± SD or *n* (%)
Age	6484	92	13.37 (1.84)
Gender	6175	401	
Male			3584 (58.0)
Female			2591 (42.0)
Grade	6551	25	
Primary school			2863 (43.7)
Secondary school			3688 (56.3)
Maternal Age	5883	693	39.07 (4.55)
Maternal Education Level	6291	285	
≤9th grade			3950 (62.8)
≤High School			1627 (25.9)
≥Undergraduate			714 (11.3)
Paternal Age	5797	779	41.63 (4.82)
Paternal Education Level	6258	318	
≤9th grade			3177 (50.8)
≤High School			2030 (32.4)
≥Undergraduate			1051 (16.8)
Ranking in class	6438	138	
1–10			1445 (22.5)
11–30			3085 (47.9)
>30			1908 (29.6)

**Table 2:** Interpersonal conflict and school connectedness across gender.

Variable	*n* (%) or Mean ± SD		*χ*^2^ or *t* Value	*p*
	Male	Female		
Quarrel with father			17.17	<0.001
No	2157 (60.6)	1690 (65.8)		
Yes	1401 (39.4)	878 (34.2)		
Father’s use of emotional punishment			7.51	0.006
No	2205 (62.0)	1678 (65.4)		
Yes	1350 (38.0)	886 (34.6)		
Father’s use of physical punishment			53.05	<0.001
No	2586 (72.7)	2073 (80.8)		
Yes	969 (27.3)	493 (19.2)		
Quarrel with mother			2.36	0.124
No	1329 (37.3)	1009 (39.2)		
Yes	2237 (62.7)	1565 (60.8)		
Mother’s use of emotional punishment			0.72	0.398
No	1927 (54.0)	1363 (52.9)		
Yes	1640 (46.0)	1212 (47.1)		
Mother’s use of physical punishment			2.48	0.115
No	2679 (75.0)	1978 (76.8)		
Yes	891 (25.0)	598 (23.2)		
Quarrel with teachers			111.99	<0.001
No	2882 (80.7)	2341 (90.5)		
Yes	690 (19.3)	246 (9.5)		
Teachers’ use of emotional punishment			86.69	<0.001
No	2139 (60.0)	1848 (71.4)		
Yes	1429 (40.0)	739 (28.6)		
Teachers’ use of physical punishment			236.07	<0.001
No	2074 (58.0)	1984 (76.8)		
Yes	1500 (42.0)	598 (23.2)		
Quarrel with peers			103.00	<0.001
No	1297 (36.5)	1273 (49.5)		
Yes	2257 (63.5)	1301 (50.5)		
Fight with peers			591.77	<0.001
No	2062 (57.9)	2239 (86.7)		
Yes	1499 (42.1)	343 (13.3)		
Peers’ use of emotional bullying			99.78	<0.001
No	1753 (49.2)	1602 (62.1)		
Yes	1809 (50.8)	979 (37.9)		
School connectedness	13.78 (4.67)	14.02 (4.24)	−2.02	0.0434

*χ*^2^ tests were used for categorical variables, *t*-tests were used for continuous variables.

**Table 3:** Interpersonal conflict and school connectedness across grade level.

Variable	*n* (%) or Mean ± SD		*χ*^2^ or *t* Value	*p*
	Primary School	Secondary School		
Quarrel with father			164.65	<0.001
No	2024 (71.3)	2034 (55.7)		
Yes	816 (28.7)	1616 (44.3)		
Father’s use of emotional punishment			59.76	<0.001
No	1944 (68.6)	2163 (59.3)		
Yes	889 (31.4)	1485 (40.7)		
Father’s use of physical punishment			36.31	<0.001
No	2042 (72.0)	2862 (78.5)		
Yes	795 (28.0)	786 (21.5)		
Quarrel with mother			228.61	<0.001
No	1366 (48.0)	1089 (29.7)		
Yes	1479 (52.0)	2577 (70.3)		
Mother’s use of emotional punishment			49.17	<0.001
No	1666 (58.6)	1827 (49.8)		
Yes	1179 (41.4)	1840 (50.2)		
Mother’s use of physical punishment			78.67	<0.001
No	1993 (70.1)	2921 (79.6)		
Yes	852 (29.9)	749 (20.4)		
Quarrel with teachers			90.55	<0.001
No	2551 (89.5)	2974 (80.9)		
Yes	301 (10.5)	703 (19.1)		
Teachers’ use of emotional punishment			30.49	<0.001
No	1946 (68.4)	2271 (61.8)		
Yes	901 (31.6)	1406 (38.2)		
Teachers’ use of physical punishment			3.74	0.0533
No	1907 (66.9)	2377 (64.7)		
Yes	942 (33.1)	1300 (35.3)		
Quarrel with peers			17.95	<0.001
No	1091 (38.5)	1602 (43.8)		
Yes	1741 (61.5)	2060 (56.2)		
Fight with peers			87.85	<0.001
No	1796 (63.1)	2712 (73.9)		
Yes	1049 (36.9)	956 (26.1)		
Peers’ use of emotional bullying			26.91	<0.001
No	1441 (50.7)	2099 (57.2)		
Yes	1400 (49.3)	1572 (42.8)		
School connectedness	15.41 (4.02)	12.73 (4.53)	25.11	<0.001

*χ*^2^ tests were used for categorical variables, *t*-tests were used for continuous variables.

**Table 4:** The associations of interpersonal conflict and school connectedness with depressive symptoms, and the moderation effects of gender and grade level.

Variable	Depressive Symptoms, *β* (95% CI)		
	Model 1	Model 2	Model 3
Quarrel with father (X)			
No	Ref	Ref	Ref
Yes	1.543 (1.275, 1.810) ***	1.449 (0.635, 2.263) ***	2.060 (1.133, 2.988) ***
X × gender		0.067 (−0.480, 0.614)	
X × grade			−0.322 (−0.872, 0.228)
Father’s use of emotional punishment (X)			
No	Ref	Ref	Ref
Yes	1.797 (1.533, 2.060) ***	1.448 (0.638, 2.258) ***	2.076 (1.179, 2.973) ***
X × gender		0.248 (−0.297, 0.792)	
X × grade			−0.176 (−0.714, 0.363)
Father’s use of physical punishment (X)			
No	Ref	Ref	Ref
Yes	1.667 (1.363, 1.971) ***	2.036 (1.098, 2.974) ***	2.404 (1.443, 3.366) ***
X × gender		−0.271 (−0.915, 0.372)	
X × grade			−0.486 (−1.084, 0.113)
Quarrel with mother (X)			
No	Ref	Ref	Ref
Yes	1.418 (1.151, 1.686) ***	0.086 (−0.736, 0.907)	1.383 (0.525, 2.240) **
X × gender		0.939 (0.389, 1.489) ***	
X × grade			0.023 (−0.512, 0.559)
Mother’s use of emotional punishment (X)			
No	Ref	Ref	Ref
Yes	1.454 (1.196, 1.711) ***	−0.198 (−0.985, 0.589)	1.337 (0.437, 2.194) **
X × gender		1.167 (0.638, 1.696) ***	
X × grade			0.074 (−0.443, 0.592)
Mother’s use of physical punishment (X)			
No	Ref	Ref	Ref
Yes	1.677 (1.378, 1.975) ***	1.216 (0.301, 2.132) **	1.910 (0.957, 2.864) ***
X × gender		0.328 (−0.289, 0.944)	
X × grade			−0.156 (−0.756, 0.444)
Quarrel with teachers (X)			
No	Ref	Ref	Ref
Yes	1.676(1.316, 2.036) ***	1.691 (0.582, 2.800) **	2.058 (0.722, 3.394) **
X × gender		–0.012 (−0.825, 0.802)	
X × grade			−0.228 (−0.993, 0.538)
Teachers’ use of emotional punishment (X)			
No	Ref	Ref	Ref
Yes	1.594 (1.327, 1.861) ***	0.784 (−0.044, 1.611)	1.961 (1.056, 2.865) ***
X × gender		0.588 (0.018, 1.158) *	
X × grade			−0.232 (−0.776, 0.313)
Teachers’ use of physical punishment (X)			
No	Ref	Ref	Ref
Yes	1.494 (1.218, 1.770) ***	1.297 (0.457, 2.138) **	2.862 (1.963, 3.762) ***
X × gender		0.147 (−0.441, 0.734)	
X × grade			−0.872 (−1.417, −0.327) **
Quarrel with peers (X)			
No	Ref	Ref	Ref
Yes	1.586 (1.325, 1.846) ***	0.815 (−0.007, 1.637)	1.738 (0.875, 2.600) ***
X × gender		0.537 (−0.005, 1.079)	
X × grade			−0.097 (−0.618, 0.425)
Fight with peers (X)			
No	Ref	Ref	Ref
Yes	1.645 (1.349, 1.941) ***	0.923 (−0.004, 1.850)	2.531 (1.637, 3.425) ***
X × gender		0.576 (−0.128, 1.279)	
X × grade			−0.585 (−1.140, −0.029) *
Peers’ use of emotional bullying (X)			
No	Ref	Ref	Ref
Yes	1.755 (1.498, 2.013) ***	1.233 (0.435, 2.031) **	1.460 (0.620, 2.299) ***
X × gender		0.373 (–0.166, 0.912)	
X × grade			0.190 (−0.322, 0.702)
School connectedness (X)	−0.375 (−0.403, −0.346) ***	−0.240 (−0.322, −0.157) ***	−0.448 (−0.547, −0.349) ***
X × gender		−0.099 (−0.155, −0.042) ***	
X × grade			0.046 (−0.013, 0.104)

* *p* < 0.05, ** *p* < 0.01, *** *p* < 0.001. Mode 1: Controlling for adolescents’ gender, grade level, ranking in class, maternal age, maternal education level, paternal age, and paternal education level. Model 2: Model 1 + interaction term (interpersonal conflict/school connectedness × gender). Model 3: Model 1 + interaction term (interpersonal conflict/school connectedness × grade).

**Table 5:** The associations of interpersonal conflict and school connectedness with depressive symptoms across different genders and grade levels.

Variable	Depressive Symptoms, *β* (95% CI)	
	Male ^a^	Female ^a^
Quarrel with mother		
No	Ref	Ref
Yes	1.066 (0.713, 1.420) ***	1.889 (1.462, 2.317) ***
Mother’s use of emotional punishment		
No	Ref	Ref
Yes	0.995 (0.659, 1.330) ***	2.079 (1.672, 2.485) ***
Teachers’ use of emotional punishment		
No	Ref	Ref
Yes	1.404 (1.063, 1.746) ***	1.939 (1.490, 2.389) ***
School connectedness	−0.344 (−0.380, −0.308) ***	−0.427 (−0.474, −0.379) ***
**Variable**	**Depressive Symptoms, *β* (95% CI)**	
	**Primary School ^b^**	**Secondary School ^b^**
Teachers’ use of physical punishment		
No	Ref	Ref
Yes	1.862 (1.442, 2.283) ***	1.221 (0.855, 1.587) ***
Fight with peers		
No	Ref	Ref
Yes	1.782 (1.351, 2.214) ***	1.481 (1.072, 1.889) ***

* *p* < 0.05, ** *p* < 0.01, *** *p* < 0.001. ^a^ Controlling for adolescents’ grade level, ranking in class, maternal age, maternal education level, paternal age, paternal education level. ^b^ Controlling for adolescents’ gender, ranking in class, maternal age, maternal education level, paternal age, paternal education level.
